# Editorial: The expanding clinical and genetic basis of adult inherited neurometabolic disorders

**DOI:** 10.3389/fneur.2023.1255513

**Published:** 2023-07-25

**Authors:** Wladimir Bocca Vieira de Rezende Pinto, Acary Souza Bulle Oliveira, Alzira Alves de Siqueira Carvalho, Hasan Orhan Akman, Paulo Victor Sgobbi de Souza

**Affiliations:** ^1^Division of Neuromuscular Diseases, Neurometabolic Unit, Department of Neurology and Neurosurgery, Federal University of São Paulo (UNIFESP), São Paulo, Brazil; ^2^Faculdade de Medicina do ABC, São Paulo, Brazil; ^3^Columbia University Medical Center, New York, NY, United States

**Keywords:** inborn errors of metabolism, neurogenetics, inherited metabolic diseases, inherited neurometabolic disorders, next-generation sequencing, metabolomics, rare diseases

The last decade of development of Neurogenetics has brought marked changes in knowledge regarding pathophysiological mechanisms, etiopathogenesis, targeted and untargeted metabolomics (and multi-omics), diagnosis of new rare genetic diseases, and the introduction of specific therapeutic approaches (1–4). The availability of genetic diagnostic methods in the clinical routine has enabled the expansion of diagnoses of rare neurogenetic diseases and significantly reduced the time required for the definitive diagnosis of rare and ultra-rare metabolic diseases (5). The classic diagnostic approach using different levels of complexity and at least two or three tiers during the biochemical evaluation has been updated (5–7). Metabolic biomarkers have emerged as important biochemical parameters during the reverse phenotyping process of complex neurological scenarios after deep phenotyping and data generated by genetic studies from next-generation sequencing-based multigene panels or whole-exome sequencing (8–11). In other cases, in the multi-omics era, transcriptomics with RNA sequencing has appeared, becoming an alternative diagnostic pathway (12). There has, therefore, been an evident change in the diagnostic work-up process of hereditary neurometabolic diseases and new guidelines and formal recommendations (13).

Adult-onset Inherited Neurometabolic Disorders represent a great challenge for clinicians due to several reasons (14, 15). First, clinical presentation, biochemical testing, and neuroimaging aspects frequently disclose atypical findings in the adult population compared to early childhood or infancy-onset forms. Thus, adult-onset phenotypes do not represent just a “childhood illness occurring in the adult population”. Furthermore, inherited metabolic diseases are commonly considered as a diagnostic hypothesis in adults only in patients with extensive diagnostic work-up which is not conclusive, and they are rarely included in the differential diagnosis of neurological presentations in acute emergency settings. Additionally, several inherited neurometabolic disorders represent potentially treatable diseases during the early stages of the disease course and with specific therapeutic approaches available, including diet therapies, enzyme replacement therapies, pharmacological chaperones, gene therapies, small interfering-RNA-based therapies, and hematopoietic stem cell transplantation (16–21).

The difficulty in diagnosing inborn errors of metabolism in adults with neurological presentation is very well demonstrated by Ferreira et al.. This systematic review article sought to assess the diagnostic complexity in such patients, analyzing the profile of types and diagnostic rates for the different genetic mechanisms from exome and genome sequencing data. The authors used this as a reference in their interpretation and conceptual definition of hereditary metabolic diseases according to the new International Classification of Inherited Metabolic Disorders (ICIMD), which markedly expanded the groups and pathophysiological mechanisms related to metabolic dysfunction in 2021 (22). The broad systematic review based on these current concepts showed an evident departure from the classic patterns of hereditary metabolic diseases: (i) there are several metabolic disorders without specific biochemical biomarkers, only with conclusive genetic diagnosis; (ii) several metabolic diseases are not directly related to enzymatic or membrane transporter defects; (iii) many neurological conditions previously thought to be primarily neuroinflammatory or neurodegenerative disorders show evidence of primary metabolic dysfunction in their pathogenesis (14, 23, 24). This manuscript represents a major representative publication related to the new ICIMD classification and the current concepts that the main researchers and clinicians have about inherited metabolic disorders.

Muthusamy et al. presented a narrative review article addressing adult-onset Leukodystrophies. This very complex issue involves a wide range of inherited metabolic diseases and the still poorly understood interface with neurodegenerative, neurometabolic, neuroinflammatory, and neurodevelopmental pathophysiological basis. It is one of the main groups of genetically determined neurological conditions in which the most current approaches related to large-scale sequencing with the use of whole-exome sequencing (and even whole-genome sequencing) have allowed in the last decades a frank expansion of the knowledge of new genetic diseases and mechanisms of etiopathogenesis. The authors present a detailed literature review covering the main clinical and neuroradiological aspects related to adult leukodystrophies, as well as an excellent diagnostic algorithm and high-quality neuroimaging studies. This is an unmissable review article for every neurologist and neuroradiologist.

Another key manuscript is presented by Nóbrega et al. with a narrative review describing the complex clinical scenario associated with Cerebrotendinous Xanthomatosis (CTX). This autosomal recessive inherited metabolic disorder represents a potentially treatable disease that frequently mimics several neurological and multisystemic conditions. Thus, it is not a surprise that CTX is commonly underdiagnosed or most frequently lately diagnosed, especially by neurologists dealing with complex neurological phenotypes. Clinical pleomorphism and marked expressivity variability are hallmarks of CTX. A detailed review of clinical, neuroradiological, and genetic aspects makes this manuscript a reference of real interest to clinicians who wish to review and stay up to date on CTX.

Huang et al. presented an original cross-sectional and longitudinal study describing the main aspects of 19 Chinese patients diagnosed with *LAMA2*-related Limb-Girdle Muscular Dystrophy (or LGMD R23). The diagnosis and recognition of this rare autosomal recessive LGMD represents a great challenge in clinical practice, especially regarding late-onset and atypical presentations, even for most neuromuscular disorder specialists. The authors demonstrated the presence of epilepsy and motor neuropathy in a high proportion of cases disclosing the complex task related to phenotype recognition and diagnostic approaches, especially in the presence of marked extra muscular involvement, a context in which metabolic myopathies and primary mitochondrial diseases are key differential diagnoses.

It is with huge satisfaction that all the Special Guest Editors of this edition invite researchers, geneticists, neurologists, neuropediatricians, and different scholars in the field of Neurosciences to seek out the up-to-date and high-quality content presented in this special edition dedicated to the emerging area of Adult Neurometabolic Diseases.

## Author contributions

All authors listed have made a substantial, direct, and intellectual contribution to the work and approved it for publication.
